# Psychological profile and self-administered relaxation in patients with craniofacial pain: a prospective in-office study

**DOI:** 10.1186/1746-160X-9-31

**Published:** 2013-10-20

**Authors:** Christian Kirschneck, Piero Römer, Peter Proff, Carsten Lippold

**Affiliations:** 1 Department of Orthodontics, University Medical Centre of Regensburg, Franz-Josef-Strauß-Allee 11, Regensburg 93053, Germany; 2 Department of Orthodontics, University Medical Centre of Muenster, Waldeyerstraße 30, Münster 48149, Germany

**Keywords:** Craniofacial pain, Temporo-mandibular dysfunction, Patient classification, Relaxation training, Psychological features

## Abstract

**Introduction:**

The objective of this study was to evaluate the psychological profile of craniofacial pain sufferers and the impact of patient subtype classification on the short-time effectiveness of a self-administered relaxation training.

**Methods:**

One hundred unselected in-office patients (67% females) suffering from chronic facial pain and/or headache with the presumptive diagnose of temporo-mandibular disorder (TMD) completed a questionnaire battery comprising craniofacial pain perception, somatic complaints, irrational beliefs, and pain behavior and were classified into subtypes using cluster analysis. They underwent a self-administered progressive relaxation training and were re-evaluated for pain perception after 3 months.

**Results:**

Pain was mild to moderate in the majority of patients. Symptom domains comprised parafunctional activities, temporo-mandibular pain and dysfunction, fronto-temporal headache, head/neck and neck/back pain. Three patient subtypes were identified regarding symptom/dysfunction level: (i) low burden (mild/moderate), (ii) psychosocial dysfunction (moderate/high), (iii) adaptive coping (moderate/mild). Self-rated adherence to the recommended relaxation training was moderate throughout the sample, but self-rated relief was significantly different between clusters. At follow-up, pain intensity was significantly decreased in all patients, whereas pain-related interference was improved only in dysfunctional and adaptive patients. Improvement of symptom domains varied between clusters and was most comprehensive in adaptive patients.

**Conclusions:**

In conclusion, craniofacial pain sufferers can be divided in meaningful subtypes based on their pain perception, irrational beliefs, and pain behaviour. A self-administered relaxation training generally yielded positive effects on pain perception, however the benefit may be greater in patients with more marked symptom impact (both dysfunctional and adaptive).

## Introduction

Orofacial pain is a common problem and frequently related to temporo-mandibular dysfunction (TMD) or generalized musculoskeletal pain [1-4]. Head-neck pain distinct from that in the masticatory muscles is among the leading accessory signs in patients with myogenic cranio-mandibular dysfunctions [5-8]. The causes of these disorders are poorly understood, however female gender and psychosocial dysfunction have been consistently reported as significant risk factors in the development and maintenance of persistent craniofacial pain [5,9-13]. Many pain problems presented to the dentist respond to treatment or resolve on their own. However, pain and disability significantly influenced by psychosocial factors are unlikely to improve substantially with conventional dental treatment alone [14,15]. When psychosocial factors are involved but not recognized, craniofacial pain may become chronic and set up a revolving door, with the patient visiting numerous providers in the wake of treatment failures that may even exacerbate patient distress [16]. Thus, the well-supported bio-psychosocial perspective on chronic pain has been accepted widely [14,17-19] and should be integrated into the dental curriculum [20]. According to this model, a patient’s experience of pain and treatment responses are affected not only by nociceptive processes, but also by factors such as mood, beliefs and appraisals as well as by social responses of others. Since, therefore, chronic pain sufferers are not a psychologically homogeneous group [19,21-24], treatment outcome depends upon patient subtype profiles [25-30].

A wide range of therapeutic approaches to craniofacial pain has been proposed, including physical therapies, drugs, and bio-behavioral modalities [31-33]. Even though a dentist is seen by the majority of patients, many of them receive additional non-dental treatment such as relaxation training [31,32,34]. The rationale is that pain and oral parafunctions may be related to stress, poor muscle discrimination or unconscious bracing of the orofacial muscles [35]. Derived from behavioral psychology, relaxation treatment aims to break existing muscle tension pain cycles and, in addition, to provide the patient with a method for controlling pain [36]. Thus, relaxation may be used as a coping strategy to be applied in everyday situations rather than in a treatment setting. Consequently, relaxation is among the most commonly used self-care techniques in chronic pain sufferers with myofascial TMD [37,38].

The present study aims to classify unselected in-office patients with persistent craniofacial pain for their self-perceived pain experiences and psychological profiles (irrational attitudes, coping behavior) and to evaluate the short-term outcomes of a self-administered relaxation training in the patient subtypes identified.

## Materials and methods

### Patients

The study group comprised 100 craniofacial pain sufferers between 20 and 75 years of age with a suspected diagnosis of TMD. Volunteers from general dental offices who gave their informed consent were randomly entered into the study in the order of their appointments. In order to ensure patient anonymity, questionnaire data were not linked to clinical records. The research was conducted in accordance with the declaration of Helsinki and the ethical regulations of the University of Regensburg, Germany.

### Questionnaires

The questionnaire battery comprised the components shown in Table 1.

**Table 1:** Questionnaires used in the study

**Items/Subscales**		**Items**	**rating scale**
**Pain inventory**			
*General items:*			
PI-A1	Item: *intensity of pain*	1	1 (not noticeable) – 6 (unbearable)
PI-A2	Item: *frequency of pain*	1	1 (daily) – 4 (once in a fortnight)
PI-A3	Item: *duration of pain*	1	1 (less than 1 hour) – 7 (permanently)
PI-A4	Item: *pain-related interference*	1	1 (unrestricted) – 5 (no activities)
*Subscales:*			1 (not at all) – 4 (very much)
PI-1	Subscale: “*parafunctional activities (PFA)*”	7	
PI-2	Subscale: “*temporomandibular dysfunction* (*TMD)*“ chewing difficulties, myofascial pain, temporomandibular pain and stiffness	8	
PI-3	Subscale: “*cervicodorsal pain* (*CDP*)”	5	
PI-4	Subscale: “*occipitocervical pain (OCP)*” including TMJ sounds	5	
PI-5	Subscale: “*frontotemporal pain (FTP)*” including migraine-like headache	7	
**McGill pain questionnaire**			1 (not at all) – 4 (very severely)
MPQ-A	subscale: *affective* descriptors	14	
MPQ-S	subscale: *sensory* descriptors	10	
**Zerssen complaint list**		24	1 (not at all) – 4 (severely)
**Irrational attitudes questionnaire**			0 (strongly disagree) – 5 (completely agree)
IA-1	“*negative self-appraisal*” (of own person and abilities)	8	
IA-2	“*dependency*” (of own behavior upon approval by others)	8	
IA-3	“*internal attribution*” (of problems to own responsibility	7	
IA-4	“*irritability*” (due to external stressors)	7	
**Pain behavior questionnaire**			1 (strongly disagree) – 5 (completely agree)
PB-1	“*avoidance*” (of social contact, leisure and work activities)	8	
PB-2	“*cognitive control*” (of pain using relaxation, attention and mind techniques)	8	
PB-3	“*social support*” (perceived by patient)	6	
PB-4	“*activity*” (in social and professional areas)	7	

The Pain Inventory (PI) involves items that were adopted from an existing clinical ad-hoc questionnaire to assess the type and extent of cranial and facial pain during the last fortnight. General pain ratings (PI-A1 to -A4) were obtained for current intensity, frequency (per week), duration (hours per day) and pain-related interference. The main questionnaire body included of a total 28 items on five factor-analytic derived subscales (PI-1 to PI-5) with sufficient internal consistency (Cronbach’s *α* ranging from 0.77 to 0.87).

The German version [39] of the McGill Pain Questionnaire MPQ was used to assess the affective (MPQ-A) and sensory (MPQ-S) dimensions of pain perception.

Zerssen’s Complaint List (CL) is a well-proven German self-assessment instrument involving 24 somatic complaints such as nausea, shortness of breath, muscle pain, and psychosomatic symptoms such as irritability, sleep disorder and uneasiness [40].

The Irrational Attitudes Questionnaire comprises a total of 30 items on 4 subscales with good reliability and validity [41].

The Pain Behavior Questionnaire [42] includes 29 items related to pain and coping behaviors classified into 4 subscales with internal consistencies ranging between α = 0.68 and 0.84 in rheumatic and headache patients.

### Treatment

Patients were provided with an audio tape containing instructions for progressive muscle relaxation after Edmund Jacobson. The following areas of systematic tensing and relaxing of muscle groups were included in subsequent order:

1. both hands and arms,

2. face (including the jaw muscles), neck and shoulders

3. chest, back and belly

4. both thighs, shanks and feet.

The participants were advised to listen to and follow the exercises as often as possible. After 3 months, a follow-up assessment was carried out including the Pain Inventory and the McGill Pain Questionnaire to allow for evaluation of the treatment effects. Moreover, patient satisfaction and compliance with the relaxation training as well as pain-relief was assessed using graded rating scales.

### Statistical analysis

Statistical data analysis using SPSS for Windows software included descriptive statistics of variable distributions, Ward’s cluster analysis of subjects followed by k-means procedures, and significance testing for between-group (Kruskal-Wallis H test) and pre-post-treatment effects (Wilcoxon signed ranks test). The general significance level was set to α < 0.05.

## Results

### Pretreatment findings

#### *General patient features*

Cluster analysis involving all questionnaire scales yielded a classification of 98% of the interviewees into three significant subgroups comprising 27 (I), 28 (II) and 43 (III) subjects respectively.

Sixty-six interviewees were females, thirty-two were males, while gender was not specified in two cases. The female-to-male ratio was 1.5 in cluster I, 3.7 in cluster II and 1.9 in cluster III, yet not significantly different. Most of the interviewees were married (47%) or coupled (18%), while 27% were single and 5% were divorced.

Patient age ranged from 20 to 75 years (mean ± standard deviation 37.7 ± 13.7 years), the interquartile range was from 28 to 46 (median 32 years). The duration of illness was 6.7 ± 6.9 years on average. The pain symptoms had existed for 6 month to 20 years (median 5 years) in 90% of subjects.

**Figure 1:**
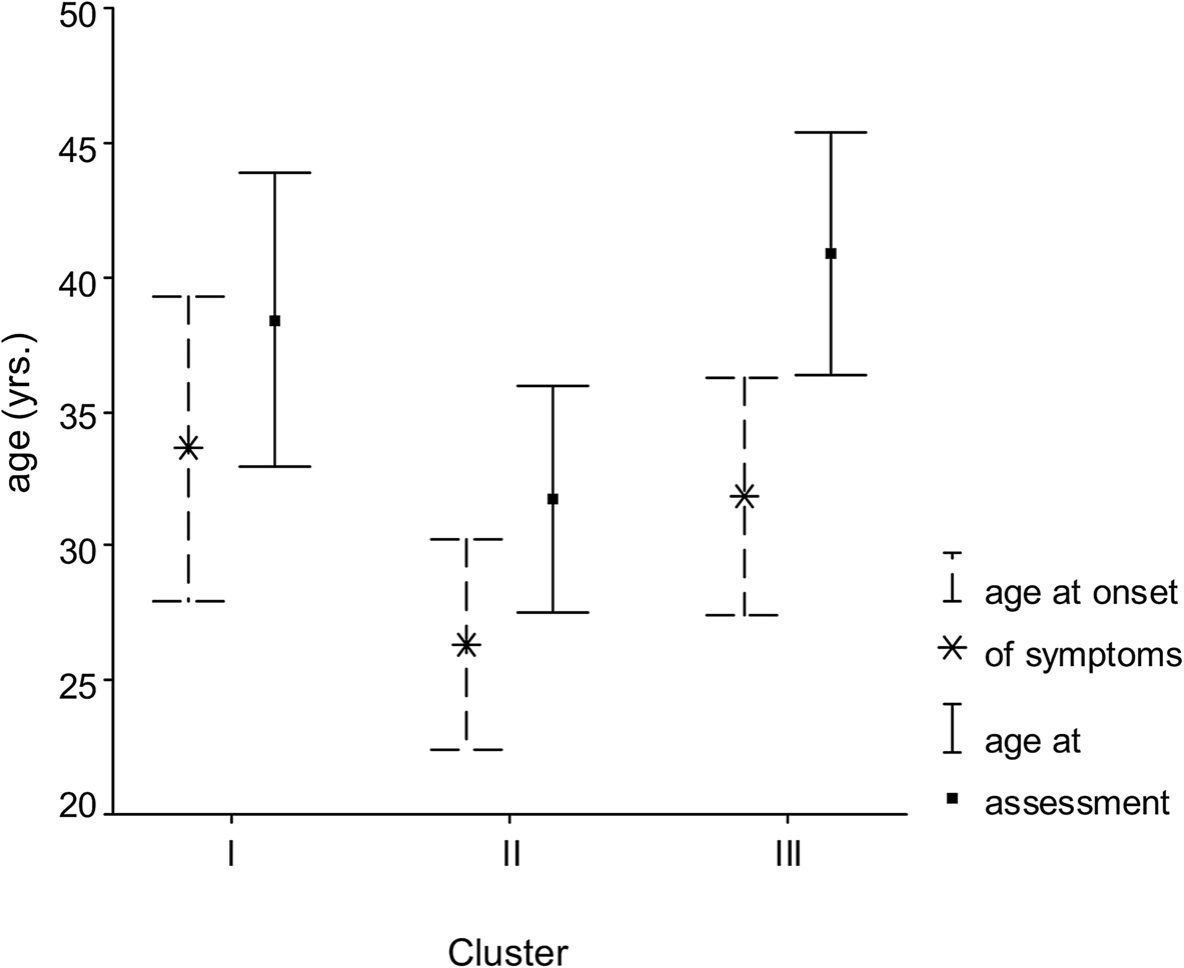
Patient age at assessment (solid line) and at reported onset of symptoms (broken line).

Patient age and duration of illness (Figure 1) were significantly different between clusters (p ≤ 0.02). Mean age was 38.4 years in cluster I, 31.7 years in cluster II, and 40.9 years in cluster III. The pain symptoms had existed for an average of 4.25 (I), 5.5 (II) and 8.9 (III) years respectively.

#### *Pain scales*

Pain intensity (PI-A1) was rated as “mild” by 35%, “moderate” by 36% and “severe” by 16% of the subjects. Pain was “not noticeable” or “very severe” in 4% respectively and “intolerable” in 2 cases. Pain intensity was significantly higher in clusters II and III compared to cluster I.

The frequency of pain episodes showed a modal value of “1-3 times a week” (36%), followed by “once in a fortnight” (26%). Daily pain episodes were reported less frequently (14%) by the interviewees, while 19% stated that they experienced pain 4-6 times a week.

The pain episodes lasted for “less than 1 hour” or “about 1-6 hours” in 34% respectively. A longer duration was stated by 28%, *i.e.* “about 6-8 hours” (14%), “about 12-24 hours” (12%) and “continuously” (2%).

Also, interference of pain with daily life activities was assessed. All activities were possible in 77% of the interviewees (“pain noticed when attended to” 24%, “pain temporarily disregarded” 26%, “pain noticed during all activities” 27%). Due to pain intensity, 11% were “restricted to light activities” and 8% were “restricted from any activities”. Pain-related interference was significantly greater in clusters II and III compared to cluster I.

The overall parameters for the Pain Inventory and McGill Pain Questionnaire items broken down by cluster membership are summarized in Table 2.

**Table 2:** Pretreatment findings for pain scales: Md median, IQR interquartile range, p significance of inter-cluster differences (Kruskal-Wallis H-test)

	**Cluster I**		**Cluster II**		**Cluster III**		**p**	**Total**	
	**Md**	**IQR**	**Md**	**IQR**	**Md**	**IQR**		**Md**	**IQR**
PI-A1	2.0	2.0; 3.0	3.0	2.0; 4.0	3.0	2.0; 3.0	0.02	3.0	2.0; 3.0
PI-A2	3.0	3.0; 4.0	2.0	2.0; 3.0	3.0	2.0; 4.0	ns	3.0	2.0; 4.0
PI-A3	1.0	1,0; 2.0	2.0	2.0; 3.0	2.0	1.0; 3.0	ns	2.0	1.0; 3.0
PI-A4	2.0	1.0; 3.0	3.0	2.0; 4.0	3.0	2.0; 3.0	0.009	2.0	1.5; 3.0
PI-1	2.0	1.5; 2.5	2.0	2.0; 3.0	2.0	1.5; 2.5	ns	2.0	1.5; 2.5
PI-2	1.5	1.0; 2.0	1.5	1.5; 2.0	1.5	1.0; 1.5	0.001	1.5	1.0; 2.0
PI-3	1.5	1.0; 2.0	2.0	2.0; 2.5	2.0	1.5; 3.0	0.002	2.0	1.5; 2.5
PI-4	2.0	1.0; 2.0	2.0	1.5; 2.5	2.0	1.5; 3.0	ns	2.0	1.5; 2.5
PI-5	1.5	1.0; 2.0	2.0	1.5; 2.5	2.0	1.0; 2.0	0.005	2.0	1.5; 2.0
MPQ-A	1.5	1.0; 1.5	2.0	1.5; 2.5	1.5	1.0; 2.0	<0.001	1.5	1.0; 2.0
MPQ-S	1.5	1.0; 1.5	1.5	1.5; 2.0	1.5	1.0; 1.5	0.04	1.5	1.0; 2.0

The overall item medians for symptom type and location were 1.5 (corresponding to “not at all” or “a little”) for TMD and 2.0 (corresponding to “a little”) for the other symptom subscales (PI-1, PI-3 to PI-5), respectively.

Patients of cluster II displayed significantly higher values for TMD (PI-2) and fronto-temporal headache (PI-5) compared to the other clusters. CDP (PI-3) was significantly less marked in cluster I compared to the clusters II and III.

The total item medians for affective (MPQ-A) and sensory (MPQ-S) pain perception were 1.5 (corresponding to “not at all” and “a little”). Both affective and sensory scores were significantly elevated in cluster II subjects.

#### *Other scales*

The overall item median of Zerssen’s Complaint List was 1.5 (corresponding to “not at all” or “scarce”). Scores were significantly higher in clusters II and III compared to cluster I.

Regarding the Irrational Attitudes subscales, the item medians were highest (2.5) for “dependency” (IA-2), lowest (1.5) for “negative self-appraisal” (IA-1) and moderate (2.0) for “internal attribution” (IA-3) and “irritability” (IA-4), respectively. All subscale scores were highest in cluster II and lowest in cluster III. These differences were significant.

The item medians of the Pain Behavior subscales were 3.5 for “activity” (PB-4), 3.0 for “cognitive control” (PB-2), and 2.0 for “avoidance” (PB-1) and “social support” (PB-3), respectively. The Pain Behavior scores were not significantly different between clusters except for “avoidance” (PB-1), which was elevated in cluster II subjects.

The overall item parameters for the Complaint List, the Irrational Attitudes Questionnaire and the Pain Behavior Questionnaire are given in Table 3.

**Table 3:** Pretreatment findings for additional scales: Md median, IQR interquartile range, p significance of inter-cluster differences (Kruskal-Wallis H-test)

	**Cluster I**		**Cluster II**		**Cluster III**		**p**	**Total**	
	**Md**	**IQR**	**Md**	**IQR**	**Md**	**IQR**		**Md**	**IQR**
CL	1.5	1.0; 1.5	2.0	1.5; 2.5	2.0	1.5; 2.0	<0.001	1.5	1.5; 2.0
IA-1	1.5	1.0; 2.5	3.5	2.5; 4.0	1.0	0.5; 1.0	<0.001	1.5	1.0; 2.5
IA-2	3.0	2.5; 3.5	3.5	3.0; 4.5	1.0	1.0; 2.0	<0.001	2.5	1.5; 3.5
IA-3	2.5	2.0; 3.0	3.5	3.0; 4.0	1.0	0.5; 1.5	<0.001	2.0	1.0; 3.0
IA-4	2.5	2.0; 3.0	3.0	2.5; 4.0	1.5	1.0; 1.5	<0.001	2.0	1.5; 3.0
PB-1	2.0	1.5; 2.0	2.5	2.0; 3.5	2.0	1.5; 3.0	0.001	2.0	1.5; 2.5
PB-2	3.0	2.5; 4.0	2.5	2.0; 3.0	2.5	2.0; 3.5	ns	3.0	2.0; 3.5
PB-3	2.0	1.5; 3.0	2.0	1.0; 2.5	2.0	1.5; 3.5	ns	2.0	1.5; 3.0
PB-4	3.5	3.0; 4.0	3.0	2.5, 3.5	3.5	3.0; 4.0	ns	3.5	3.0; 4.0

### Posttreatment findings

**Figure 2:**
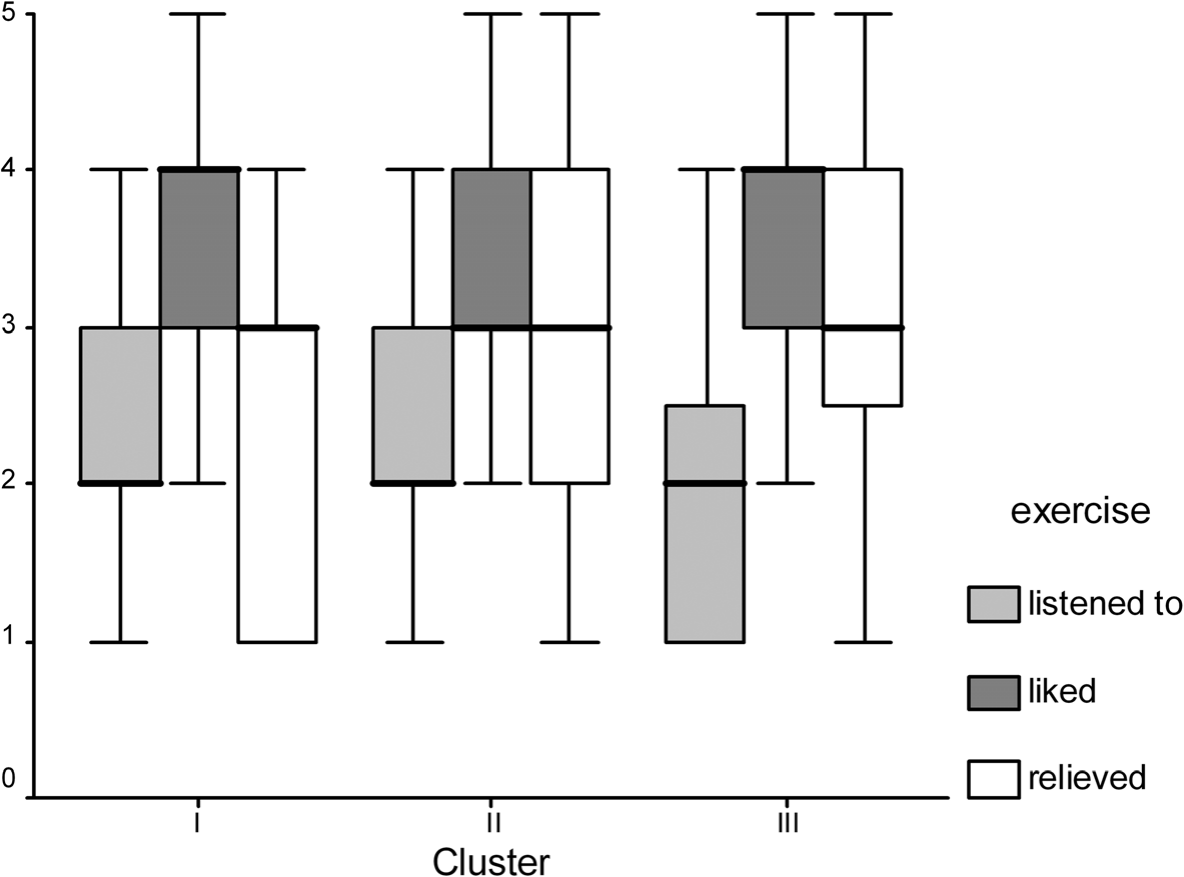
Patient appraisal of relaxation exercise.

Patient-perceived pain was re-evaluated 3 months after instruction for the self-administered relaxation training. While acceptance of (median 4 “much”) and adherence to (median 2 “1-3 times a week”) the program were not significantly different between clusters, self-rated improvement varied between cluster I patients (median 2.5) and cluster II and III patients (median 3.0). Thus, the clusters were significantly (p = 0.01) different for self-assessed pain relief after relaxation exercise (Figure 2).

Pre-post analyses of the pain-related scales (H-Test) confirmed global improvements for the total sample, however revealed significant differences between clusters for pain intensity (PI-1 p = 0.03), episode duration (PI-3 p = 0.01), pain-related interference (PI-4 p = 0.008), the affective (MPQ-A p = 0.04) and sensory (MPQ–S p = 0.005) components of pain perception as well as the item scores of TMD (PI-A2 p < 0.001 and FTP (PI-A5 p < 0.001).

Pain intensity (PI-A2) significantly decreased in all patient irrespective of cluster membership (p ≤ 0.005). The frequency (PI-A3) and duration (PI-A4) of pain episodes as well as pain-related interference (PI-A5) were significantly reduced in cluster II and III patients (p ≤ 0.025), but not in Cluster I patients. Differential effects were observed for the domains of the Pain Inventory. Cluster I patients showed significant improvements of CDP (PI-3) and FTP (PI-5) and either dimension of the MPQ. In cluster II, significant improvement was achieved for TMD (PI-2) and OCP (PI-4). The most global improvements were revealed for cluster III patients (all scales except TMD). The results are given in Table 4.

**Table 4:** Posttreatment findings for pain scales: Md median, IQR interquartile range, p significance of pre-posttreatment differences (Wilcoxon signed-rank test)

	**Cluster I**			**Cluster II**			**Cluster III**			**Total**		
	**Md**	**IQR**	**p**	**Md**	**IQR**	**p**	**Md**	**IQR**	**p**	**Md**	**IQR**	**p**
PI-A1	2.0	2.0; 3.0	0.005	3.0	2.0; 3.5	0.003	2.5	2.0; 3.0	<0.001	2.5	2,0; 3.0	<0.001
PI-A2	3.0	3.0; 4.0	ns	2.5	2.5; 3.0	0.003	3.0	2.5; 4.0	0.005	3.0	2.5; 4.0	<0.001
PI-A3	1.5	1.0; 2.0	ns	2.0	2.0; 3.0	0.008	2.0	1.0; 3.0	0.025	2.0	1,0; 3.0	<0.001
PI-A4	2.0	1.0; 3.0	ns	3.0	2.0; 3.5	0.005	2.5	2.0; 2.5	<0.001	2.0	1.5; 3.0	<0.001
PI-1	2.0	1.5; 2.5	ns	2.0	1.5; 2.5	ns	2.0	1.5; 2.0	0.002	2.0	1.5; 2.5	<0.001
PI-2	1.5	1.0; 1.5	ns	1.5	1.5; 2.0	0.004	1.5	1.0; 1.5	ns	1.5	1.0; 2.0	0.001
PI-3	1.5	1.0; 2.0	0.046	2.0	2.0; 2.5	ns	2.0	1.5; 2.0	0.001	2.0	1.5; 2.0	<0.001
PI-4	2.0	1.0; 2.0	ns	2.0	1.5; 2.5	0.046	2.0	1.5; 2.0	0.002	2.0	1.5; 2.0	<0.001
PI-5	1.5	1.5; 2.0	0.042	2.0	1.5; 2.5	ns	1.5	1.5; 2.0	0.04	1.5	1.5; 2.0	0.008
MPQ-A	1.0	1.0; 1.5	0.01	2.0	1.5; 2.5	0.04	1.5	1.0; 2.0	ns	1.5	1.0; 2.0	0.002
MPQ-S	1.5	1.0; 1.5	0.02	1.5	1.5; 2.0	ns	1.5	1.0; 1.5	ns	1.5	1.0; 1.5	0.003

## Discussion

The multifactorial model of TMD and orofacial pain has prompted numerous studies that emphasize the psychological factors of these disorder and involve alternative treatment approaches [18,31-33]. However, results from dental in-office studies are largely lacking, although most patients have no access to special care institutions.

In order to avoid selection bias, eligible patients with chronic craniofacial pain from general dental practices were consecutively entered into the study and anonymously completed the questionnaire battery. Questionnaires are a well-tried instrument for the assessment of subjective experiences and psychological characteristics in chronic pain [43].

### General findings

The sociodemographic characteristics of our sample suggest a 2:1 ratio with females prevailing and a main age range between 28 and 46 years. This distribution corresponds to reports from literature where the highest prevalence of facial pain has been found in females during the main reproductive age from 25 to 40 years [2,5,12,44-46]. Since associated pain areas are also more frequently reported by females [1,47], an increased sensitivity to pain in females has been suggested to account for these findings [10,46].

Almost three thirds of the subjects rated their pain as mild or moderate, while only a minor part reported severe pain and/or daily occurrence of pain which largely prevented them from everyday activities. These findings agree with reports from literature implying that orofacial pain is mild to moderate in 75% of the cases and causes only slight restrictions of everyday activities. Von Korff [48] found more marked restrictions in 16% for facial pain and 30% for headache, while 30-40% of the patients showed the lowest grade of pain intensity. The burden of facial pain assessed in German population surveys corresponds well to our results [44].

Simultaneous occurrence of facial and cranial pain at variable locations has been frequently reported in literature [5-7,49]. Moreover, patients with chronic craniofacial pain frequently show a comorbidity of pain in other regions of the body, especially the neck and back, or other functional complaints which are rarely reported to the dentist [1-4]. Therefore, we utilized Zerssen’s CL to assess the total burden of complaints which was in the abnormal range in more than 90% of the interviewees.

Perceived pain intensity, pain-related interference with daily activities, and behavioral treatment outcome have been shown to be associated with dysfunctional attitudes [50,51]. The frequency of maladaptive, irrational beliefs is increased particularly in patients with facial pain of unknown origin, myofascial pain and daily headache compared to patients with other TMD diagnoses [9,52]. The present unselected sample displayed moderate overall scores which may reflect the relatively low symptom burden and the use of generalized attitudes instead of pain-specific beliefs. Negative “irrational” beliefs are associated with rather passive pain behaviors such as avoidance, inactivity, and social support-seeking [53]. It is little surprising, hence, that our findings involving pain behavior were generally less dysfunctional as compared to other studies [37,54]. In fact, agreement with statements indicating activity and cognitive control was significantly stronger than with those involving social support-seeking and avoidance.

### Treatment response

Self-care and home exercise regimens in orofacial pain sufferers have been encouraged by several studies, all the more so as they are simple and non-invasive, have a favourable cost benefit ratio over other treatment modalities, allow an easy self-management approach and can be managed by the general dental practitioner [14,38,55-57]. However, the provider should not simply hand the patient an audiotape and expect him to benefit from listening to the tape [58]. Rather, structured audiotape programs on relaxation have been shown to be effective when the patient was first instructed on their use and encouraged to use them [59]. In the present study, therefore, craniofacial pain sufferers were provided with advice and instructions about the progressive muscle relaxation program to be followed. Acceptance of the program was rather uniform among patients. Similarly, a significant global benefit was shown for the whole sample. In agreement with this finding, the effectiveness of relaxation techniques in the management of TMD including headache, facial and musculoskeletal pain has been proven in several systematic reviews and meta-analyses [33,60-63]. Programs involving relaxation techniques to relieve pain may even be superior to that of occlusal splints in persons with acute or chronic myofascial or muscular TMD [62,64].

### Psychological subtyping

The acknowledgement of the interaction between somatic complaints and psychosocial factors warrants a multidimensional approach to the classification of patients with craniofacial pain and dysfunctions. Two decades ago, Turk and Rudy [21] suggested an empirical taxonomy of chronic pain patients comprising three psychophysical subgroups that have been referred to as “dysfunctional”, “interpersonally distressed” and “adaptive copers”. They were replicated for low back pain, headache, and TMD [22]. Later, “repressive patients” were suggested to form a subtype of the “dysfunctional” patients’ [65]. Dysfunctional patients show high pain intensity, marked pain interference with occupational and social activities, affective distress and reduced physical activity, whereas “adaptive” patients judge their pain as less intense and interfering, are emotionally less distressed and report more activity and more control of their lives [18,21,22,54]. Other studies arrived at similar classifications of temporo-mandibular pain sufferers who were different in their illness beliefs, affective involvement, coping mechanisms and impact on private and professional life, but not in their objective physical findings [19,23,24]. Although meaningful subgroups of pain sufferers were consistently and reproducibly established, only few studies aimed to predict differential treatment outcome from this classification [25-30,66].

In our study, homogeneous patient clusters were identified on the basis of the whole body of questionnaire data assessed. In agreement with previous studies [19,21,22,26,66] three patient subtypes emerged which were significantly different for pain parameters and psychological profile. Cluster I subjects on average showed the lowest symptom burden as reflected by the Pain Inventory and Zerssen’s Complaint List. The irrational attitudes scores of these subjects are between those of the two other clusters. The increased use of cognitive control and the low avoidance scores may be related to the lower subjective pain stress. Thus, this subgroup resembles the type of patient referred to in literature as “uncomplicated” [19], “low impact” [26] or “good pain control” [66] with low levels of pain, impairment and dysfunction.

Overall self-perceived pain relief after relaxation exercise was significantly lower in cluster I patients compared to the two other clusters. This was supported by the pre-post treatment findings for pain ratings. The low-symptom Cluster I patients showed the least pre-posttreatment differences. Perceived pain intensity significantly decreased like in the other groups, but pain duration, frequency and interference which were already low pre-treatment did not change. Significant improvements were also observed for the pain domains CDP and FTP and either dimension of the MPQ.

Cluster II comprised persons of younger age whose pain symptoms prevail in the fronto-temporo-mandibular region and were perceived as more severe and emotionally distressing than on average. This is consistent with findings indicating that the preference of MPQ pain descriptors is associated with pain chronicity [67] as well as pain type and localization [68]. In contrast to other TMD sufferers, patients with primarily myogenic facial pain and headache choose more affective descriptors and rate the pain as more intensive [24,52,67,68]. The higher psychosomatic burden reflected by the CL scores underpins the picture of subjects who display various pain locations of similar intensity and a number of accessory complaints. Irrational beliefs, especially a conspicuously negative self-appraisal were consistently more marked in cluster II as compared to the two other groups. Moreover, these persons experienced less social support and responded with increased withdrawal instead of activity. This may partly be due to increased sensory perception of primarily fronto-temporal pain. In summary, the negative self-image, the reduced interpersonal satisfaction, the psychosomatic burden and the affective pain component may point to premorbid affective lability and introversion. Thus, this subgroup is characterized by increased psychological maladaptation and corresponds well to the “chronic pain syndrome” [66] or “dysfunctional” type described in literature [19,21,22,69]. These patients showed significant general reductions of pain intensity, frequency, duration, and interference. Moreover, the temporo-mandibular and occipito-cervical pain domains were significantly improved as well as the affective dimension of the MPQ.

Interviewees belonging to group III made up nearly half of our sample. Their symptoms had existed for 9 years on average and, thus, about double as long as in the other groups. Pain was localized primarily in the occiput, neck and back areas and partly accompanied by parafunctional activities and a higher burden of somatic complaints in comparison to cluster I. In contrast, the frequency of fronto-temporal headache and particularly of characteristic TMD signs was reduced. Pain intensity was nearly as high as in group II. Members of group III showed the highest degree of psychological normality as reflected by the Irrational Attitudes Questionnaire. Pain behavior resembled that of group I involving activity as the prevailing coping strategy. More social support was reported in comparison to the other groups. Thus, group III is primarily characterized by active coping with pain, representing the “adaptive” type of patients [19,21,66,69]. Alternatively, such patients may reflect a “stoic” profile which was described for persons with a long history of pain who show little psychological dysfunction relative to perceived pain intensity and pain-related interference [70,71]. Presumably, a stable primary personality and long-lasting “adaptation” to the burden of complaints may have led to successful coping in most of these individuals. In some patients, however, a mismatch between pain perception and psychological self-appraisal may result from a type of “Freudian repression” rather than adaptation. While pain intensity and somatic complaints are similar to those of “dysfunctional” patients, the mild cognitive symptoms mimic those of “adaptive copers” [65].

Posttreatment, cluster III patients revealed significantly reduced pain intensity, frequency, duration, and interference like cluster II patients, but pain descriptors in the MPQ remained unchanged. Furthermore, significant improvements were found for all pain domains except TMD which showed a significantly lower pretreatment level as compared to the other clusters.

All in all, the typological composition of our sample best resembles that reported by Suvinen et al. [19] who distinguished a highly distressed, psychosocially maladaptive group, a moderately distressed, behaviorally functional group, and a predominantly physical disorder group with an unremarkable psychosocial profile. Moreover, there were cluster-specific differences in the extent of change for general pain intensity and interference, the pain domains PFA, OCP and CDP, and the MPQ descriptor ratings. The lesser improvement of pain frequency, duration and interference observed in cluster I patients may be accounted for by lower baseline levels of these variables. However, significant effects were found for general pain intensity ratings and selected pain domains. Similarly, significant reductions in pre-to-posttreatment pain were found in the “low impact” subgroup of migraine headache sufferers [26]. Thus, any craniofacial patient subtype may benefit from a self-administered relaxation training. It may be applied even prior to in-depth diagnosis, in cases with unclear etiology, or in addition to conservative treatment. Nevertheless, the variable treatment outcomes observed support the call for tailored treatment for psychologically defined subgroups or individual patients with chronic pain [14,56,72,73].

## Conclusions

• Craniofacial pain sufferers may be classified into subtypes with different symptom-related and psychosocial characteristics (low symptom and psychosocial impact – high symptom and psychosocial impact – high symptom impact, low psychosocial impact).

• A self-administered relaxation training, which is cost-effective and time-saving, generally yields positive effects in patients with craniofacial pain.

• Different patient subtypes benefit differently from such training, which is most promising in patient with high symptom impact and moderate or high dysfunction.

### Consent

Written informed consent was obtained from the patient’s guardian/parent/next of kin for the publication of this report and any accompanying images.

## Competing interest

The authors declare that they have no competing interests.

## Authors’ contributions

CK, PR, PP and CL contributed to the conception, design and coordination of the study. CK made substantial contributions to the acquisition of data and the preparation of the manuscript. CK drafted and wrote the manuscript. PR, PP and CL revised the manuscript. All authors read and approved the final manuscript.
